# On the Discovery and Development of Pimavanserin: A Novel Drug Candidate for Parkinson’s Psychosis

**DOI:** 10.1007/s11064-014-1293-3

**Published:** 2014-03-30

**Authors:** Uli Hacksell, Ethan S. Burstein, Krista McFarland, Roger G. Mills, Hilde Williams

**Affiliations:** ACADIA Pharmaceuticals Inc., 11085 Torreyana Road, Ste. 100, San Diego, CA 92121 USA

**Keywords:** Pimavanserin, Parkinson’s disease psychosis (PDP), 5-HT_2A_ inverse agonist

## Abstract

Parkinson’s disease psychosis (PDP) is a condition that may develop in up to 60 % of Parkinson’s patients, and is a major reason for nursing home placement for those affected. There are no FDA approved drugs for PDP but low doses of atypical anti-psychotic drugs (APDs) are commonly prescribed off-label. Only low-dose clozapine has shown efficacy in randomized controlled trials, but all APDs have black box warnings related to the increased mortality and morbidity when used in elderly demented patients. Using molecular pharmacological profiling of a large collection of marketed drugs, we discovered that potent inverse agonist activity against 5-HT_2A_ serotonin receptors was a common feature of atypical APDs, especially the atypical APDs used to treat PDP. Since low-dose clozapine therapy selectively blocks this receptor, it was hypothesized that a highly selective 5-HT_2A_ receptor inverse agonist might provide good symptom control in patients suffering from PDP, with a greatly improved safety and tolerability profile. A high throughput screening and subsequent chemical lead optimization campaign to develop potent, selective 5-HT_2A_ receptor inverse agonists was launched, eventually resulting in the discovery of pimavanserin. Pimavanserin displays nanomolar potency as a 5-HT_2A_ receptor inverse agonist, selectivity for 5-HT_2A_ over 5-HT_2C_ receptors, and no meaningful activity at any other G-protein coupled receptor. It demonstrated robust activity in preclinical models of schizophrenia and PDP, and did not worsen motoric symptoms, in contrast to the APDs tested. In a Phase III clinical trial, pimavanserin showed highly significant benefits in the primary endpoint, the scale for assessment of positive symptoms-PD, a scale adapted for use in PDP. In addition, improvements in all other efficacy endpoints, including physician’s clinical global impression, caregiver burden, night-time sleep quality and daytime wakefulness, were seen. Pimavanserin demonstrated good safety and tolerability and did not worsen motoric symptoms as assessed by the unified Parkinson’s disease rating scale parts II and III. An open-label extension study has further demonstrated that pimavanserin is safe and well-tolerated with long-term use. Pimavanserin may therefore offer a viable treatment option for patients suffering from PDP.

## Background

Parkinson’s disease (PD) is a neurodegenerative disease that affects millions of people worldwide. PD is more common in older people and the number of PD patients is expected to increase with the increasing age of the worldwide population. The most obvious symptoms of PD are motoric and include tremor, bradykinesia, rigidity, and disturbed balance and posture. The motor symptoms in PD are directly related to the progressive degeneration of primarily brain dopamine (DA) neurons. No disease modifying drugs have yet been developed for PD and various symptomatic drugs are currently used to treat the motor symptoms of the disease. These drugs include DA receptor agonists, MAO inhibitors, L-DOPA and amantadine [1].

While motor symptoms of PD used to be the focus of treatment, it has now been realized that non-motor symptoms are equally disturbing to the patient [2]. The most common non-motor symptoms include depression, sleep problems, psychosis and dementia. Parkinson’s disease psychosis (PDP) [3, 4], which is characterized by hallucinations and/or delusions, may develop in up to 60 % of PD patients [5], is persistent and progressive and associated with deterioration in quality of life as well as increased morbidity and mortality. Psychosis has been identified as the leading cause of nursing home placement among PD patients [6]. Currently, there is no effective, tolerated and safe therapy available for treatment of PDP. While low doses of clozapine are approved as a second line therapy in Europe, no first-line therapy is available and no PDP drug is approved in any other major market.

There are many marketed anti-psychotic drugs (APDs) but they all block the dopamine (DA) D_2_ receptors which are the target for the symptomatic DA replacement therapy in PD. Hence, they are pharmacologically contraindicated for PD and at effective antipsychotic doses these drugs induce intolerable motor side effects in PD patients. There is one exception: clozapine, which is used to treat schizophrenia in daily doses from 300 to 900 mg, is tolerated and effective in treating the psychosis in PDP patients when given at more than 10 fold lower doses than used in schizophrenia therapy [7, 8].

Despite the clinical evidence for efficacy and tolerability of clozapine in PDP therapy, it is infrequently used. There are two major problems with clozapine therapy in PDP: First, clozapine is not safe, even at the low doses used for PDP. It may still cause agranulocytosis [9] and, thus, requires frequent blood monitoring. Second, the histamine H_1_ antagonism of clozapine leads to sedation. This adds to the excessive daytime sleepiness seen in PD patients. Rather than using clozapine, neurologists tend to resort to using quetiapine for managing PDP [10]. Unfortunately, while low doses of quetiapine are motorically tolerated by patients, these low doses have not demonstrated efficacy [11] and still are associated with excessive sedation. In addition, like all other marketed APDs, clozapine and quetiapine have a black box warning for use in elderly demented patients with psychosis due to increased mortality and morbidity.

The low doses of clozapine which have shown efficacy in PDP are likely to selectively block only 5-HT_2A_ and H_1_ receptors and do not appear to block the brain DA D_2_ receptors [12, 13]. Since H1 receptor antagonism is known to produce sedation but is unlikely to contribute to the antipsychotic effect of clozapine, the effectiveness of low-dose therapy with clozapine in PDP suggests that 5-HT_2A_ receptor blockade is the relevant target mechanism [14]. More recent data from a PET study in patients provide additional evidence for the importance of the 5-HT_2A_ receptor in PDP by demonstrating that visual hallucinations in PD are correlated with excessive 5-HT_2A_ neurotransmission [15].

## The Discovery of Pimavanserin

In the late 1990s ACADIA scientists started a chemical genomics effort aimed at improving the understanding of the targets for drugs acting on the central nervous system [16]. A comprehensive library of marketed CNS drugs were evaluated for activity on a wide range of G-protein coupled receptors (GPCRs) using the Receptor Selection and Amplification Technology™ (R-SAT™) platform, a high-throughput functional assay technology that is well suited for chemical genomics and high-throughput screening (HTS), and is applicable to a wide array of genetic targets including most GPCRs, receptor tyrosine kinases, cytokine receptors, and nuclear receptors [17]. R-SAT™ utilizes the principles of genetic selection and is based on the observation that oncogenes and many receptors induce proliferation or transformation responses in NIH-3T3 cells. Agonists preferentially select and amplify cells that express functional receptors. In cases where the genetic target exhibits constitutive activity, cellular proliferation occurs in the absence of added agonists. In such cases, inverse agonists can be readily identified by their ability to suppress proliferative responses [18–21]. Typically, the optimal signal is observed 5–6 days post-transfection, a period of time during which the reporter is amplified in the proliferating cells and diminished in the quiescent cells [22].

GPCRs frequently possess some degree of ligand independent or constitutive activity [23]. Of the various functional assays used for HTS, RSAT™ may provide the most sensitive means of detecting constitutive activity, possibly due to its assay length of 5–6 days which allows for amplification of constitutive responses to occur. For example, a direct comparison of calcium flux, phosphatidyl inositol hydrolysis (PI) and R-SAT™ assays reveals the constitutive activity of the Ghrelin receptor is most easily detected using R-SAT™ [17]. These findings strongly agree with a previous study in which the constitutive responses of wild-type and mutant forms of the 5-HT_2A_ receptor were much more apparent using R-SAT™ assays compared with PI assays [24].

While screening numerous typical and atypical APDs, we discovered that most of the atypical APDs, including clozapine, had one activity in common which separated them from the typical antipsychotic agents. They were potent and fully efficacious inverse 5-HT_2A_ agonists [25] and they were less or much less potent as DA D_2_ receptor antagonists. The efficacy of low-dose clozapine in PDP therapy and the observation that atypical APDs appear to have several advantages over the older typical agents led to the hypothesis that selective 5-HT_2A_ inverse agonist activity might be an appropriate target mechanism to explore in a drug discovery program [14]; thus we initiated a program to discover novel 5-HT_2A_ receptor inverse agonists.

A functional HTS R-SAT™ assay for 5-HT_2A_ inverse agonists was configured by expression of the human 5-HT_2A_ human receptor in NIH 3T3 cells together with a marker gene to permit signal detection using a colorimetric method. A proprietary compound library of 130,000 chemically diverse small molecules was screened in the HTS assay at a concentration of 3 μM. Of the initial 500 hits, 100 were characterized as potent 5-HT_2A_ inverse agonists. Following further screening for selectivity and subsequent lead optimization, AC-90179 was identified as a selective 5-HT_2A_ inverse agonist [26]. It had nearly 100 fold selectivity for 5-HT_2A_ receptors compared to 5-HT_2B_, 5-HT_2C_ and 5-HT_6_ receptors as an inverse agonist. At concentrations less than or equal to 1 μM, it did not interact with other monoaminergic receptors. Although the oral bioavailability of AC-90179 was very low, it was useful for initial proof of concept studies in rodents. As expected, AC-90179 dose-dependently eliminated head twitches induced by DOI, a behavior mediated by 5-HT_2A_ receptor stimulation. Also, AC-90179 inhibited MK-801-induced but not amphetamine-induced locomotor activity. At the dose that effectively inhibited MK-801-induced locomotor activity, AC-90179 did not reduce spontaneous locomotor activity. Importantly, AC-90179 was effective in restoring prepulse inhibition (PPI) response disturbed by DOI. All these effects were expected based on previous studies describing the pharmacology of MDL-100,907, a selective 5-HT_2A_ antagonist [27, 28].

**Fig. 1:**
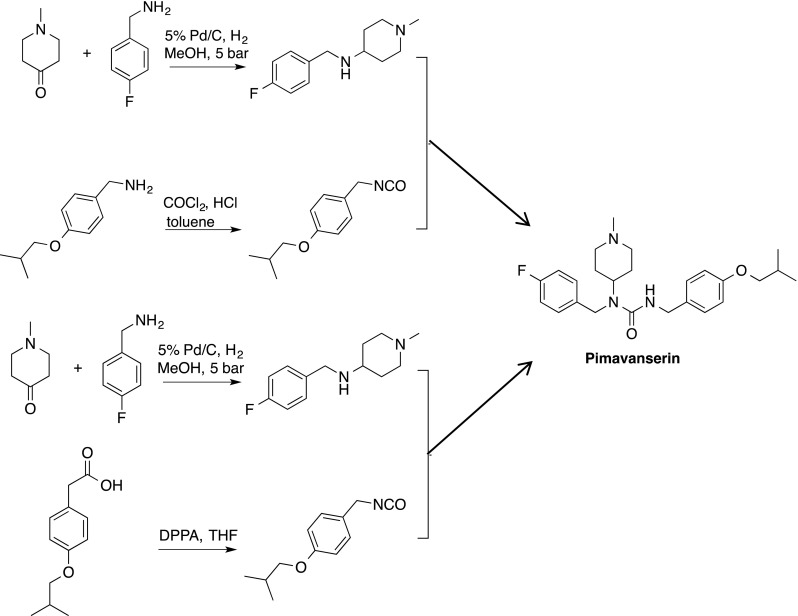
Two facile synthetic routes providing pimavanserin

**Fig. 2:**
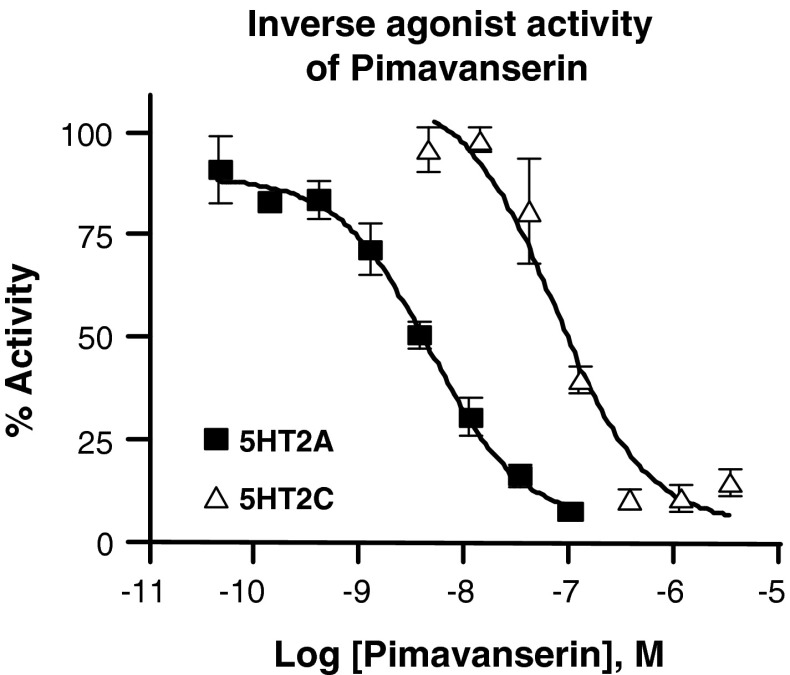
Inverse agonist activity of Pimavanserin. R-SAT™ assays were performed with 5-HT_2A_ and 5-HT_2C_ receptors as described [24] using the indicated concentrations of Pimavanserin. Inverse agonist activity was normalized to ritanserin (not shown)

The in vitro and in vivo pharmacology of AC-90179 was attractive and, therefore, a major lead optimization effort was launched to develop an orally bioavailable analogue with similar pharmacology. This effort led to the discovery of pimavanserin (ACP-103) [29], a molecule with similar structural characteristics as AC-90179 but with much greater oral bioavailability. Pimavanserin is an achiral compound which is easy to synthesize in small or large scale from readily available starting materials (Fig. 1). Pimavanserin has no structural resemblance to the APDs. Pimavanserin is a potent, selective 5-HT2A inverse agonist, with selectivity over 5-HT_2C_ receptors in binding and functional assays and little to no activity at other GPCRs in contrast to the available APDs (see Fig. 2; Table 1). Thus, the structural characteristics and pharmacological selectivity profile of pimavanserin differentiates it from typical as well as atypical APDs.

**Table 1:**
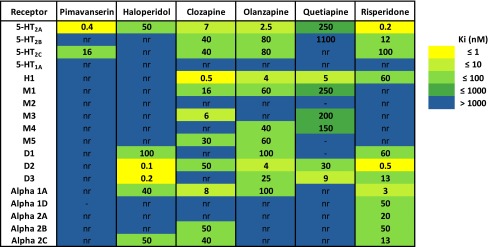
Receptor selectivity of pimavanserin compared to some antipsychotic drugs

Data are Ki values in nM derived from functional antagonist R-SAT™ assays. –,not done; *nr*, no response

Pimavanserin’s behavioral profile in rodents [29] is consistent with that of other 5-HT_2A_ antagonists like AC-90179 and MDL-100,907. Thus, it blocks DOI-induced head twitch and MK-801-induced hyperactivity. Additionally, it prevents DOI- and MK-801-induced disruptions in prepulse inhibition. These behavioral effects are seen with atypical APDs like risperidone, clozapine and quetiapine, which have appreciable antagonist activity at 5-HT_2A_ receptors. However, unlike the APDs, pimavanserin lacks DA D_2_ antagonist activity and thus does not show reliable, dose dependent blockade of amphetamine-induced activity. Hence, pimavanserin shares several, but not all of the preclinical behavioral characteristics observed with atypical APDs.

**Fig. 3:**
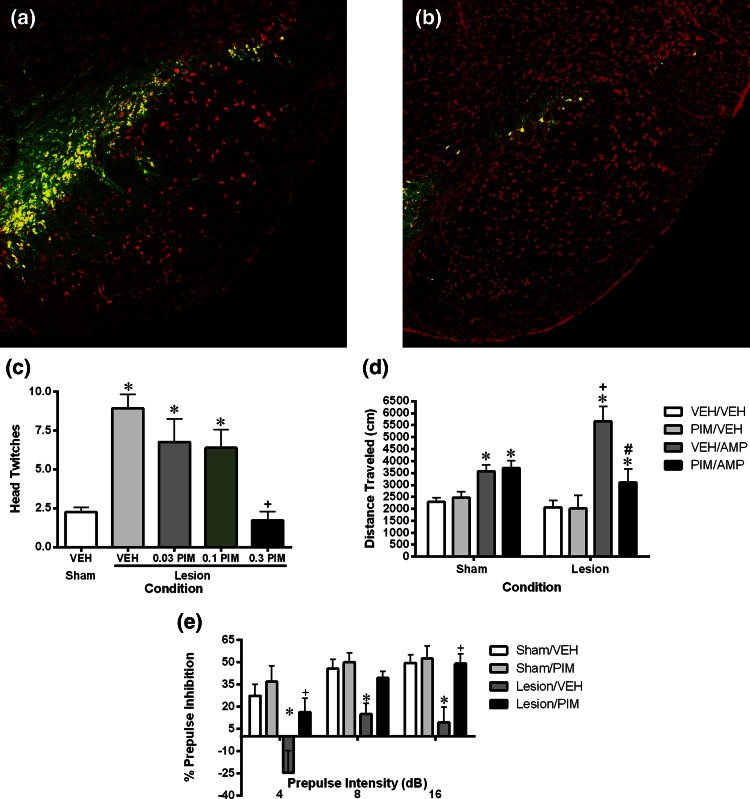
Altered tyrosine hydroxylase immunofluorescence and behavior in animals with bilateral lesions of the SN. Colocalization (*yellow*) of tyrosine hydroxylase (*green*) and the neuronal marker Neurotrace (*red*) immunostaining in a sham control (**a**) and lesioned animal (**b**). On average, lesioned animals showed a 75 % reduction in TH immunofluorescence 4 weeks after lesion. Lesioned animals displayed augmented spontaneous head twitches (**c**), augmented amphetamine-induced locomotion (**d**) and disrupted prepulse inhibition (**e**) which were all reversed by treatment with pimavanserin

In order to assess whether pimavanserin might be effective in treating PDP, a rodent model of PD was employed where rats received bilateral lesions of the substantia nigra (SN). Using this procedure, there was rapid loss (within 1 day) of tyrosine hydroxylase, a marker of healthy dopaminergic neurons, in the SN. Loss continued until a maximal loss of roughly 75 % was reached around 2 weeks after lesion (Fig. 3). Notably, following SN lesion, animals developed difficulty initiating and maintaining motor behaviors, in a manner that was reversed by treatment with L-DOPA. In addition to motor deficits, these animals also displayed a psychosis-like pattern of behavioral changes, i.e., they displayed changes in behaviors typically used to assess the efficacy of antipsychotic medications. These included increased numbers of spontaneous head twitches, augmented amphetamine-induced hyperactivity and disrupted prepulse inhibition. Notably, pimavanserin not only reversed the psychosis-like behaviors, but did so without augmenting motor problems or blocking the ability of L-DOPA to improve motor behavior (Fig. 3) [30]. The appearance of altered 5-HT_2A_-dependent behaviors in lesioned rats is consistent with data demonstrating that destruction of dopaminergic neurons in animals leads to adaptations in serotonergic signaling, including increased extracellular 5-HT, increased serotonin transporters, sprouting of serotonergic afferents to the striatum an up-regulation of 5-HT_2A_ mRNA in the striatum [31–36].

Because the atypical APDs quetiapine and clozapine are prescribed to PDP patients, we assessed whether these compounds might also work in our animal model of PDP and compared their in vivo profiles with that of pimavanserin [37]. Specifically, we assessed the dose of pimavanserin, clozapine and quetiapine required to reduce psychosis-like rodent behaviors and the dose that caused unwanted side effects like disruption of coordinated motor behavior or sedation. In this manner a therapeutic ratio was determined. All doses of quetiapine and most doses of clozapine that were effective at blocking psychosis-like behavior in bilaterally lesioned rats impaired motor behavior. In contrast to clozapine and quetiapine, pimavanserin reduced psychosis-like behavior at doses more than a hundred-fold lower than doses that reduced locomotion, and no dose of pimavanserin blocked D_2_-dependent motor behavior. Pimavanserin’s high therapeutic ratio as assessed in these animal models suggests that it may effectively treat symptoms of PDP without the risk for concomitant loss of motor control and sedation commonly seen with available atypical APDs.

## Clinical Studies with Pimavanserin

Initial clinical studies in normal healthy volunteers showed that pimavanserin was well tolerated in humans both when given acutely and subchronically [38]. Nausea and vomiting were considered to be dose-limiting following 14 days of once daily oral dosing at 150 mg; 100 mg QD of pimavanserin given over the same period was therefore estimated to be the maximum tolerated dose. Dose proportional plasma exposure over a wide range of doses was also observed in these studies. The half-life of pimavanserin was estimated to be between 55 and 60 h and no high-fat food effect on bioavailability was observed [39].

Our rodent experiments demonstrated that pimavanserin readily crosses the blood brain barrier and acts as a CNS-active 5-HT_2A_ inverse agonist. In order to investigate what doses of pimavanserin that blocked the brain 5-HT_2A_ receptors in humans, we conducted a PET study in which the 5-HT_2A_/5-HT_2C_/D_2_ ligand 11C-NMSP was used as the radio ligand. The results showed that a dose of 10 mg of pimavanserin was able to almost completely occupy the brain 5-HT_2A_ receptors in normal healthy volunteers [40]. Because 11C-NMSP is not a selective ligand, it predominantly labels DA D_2_ receptors in the striatum. Consequently, and as expected based on the selectivity profile of pimavanserin, we observed only minimal displacement of 11C-NMSP from striatum at doses as high as 100 mg. This underlines the different pharmacology of pimavanserin compared to that of all other APDs which interact with DA receptors and numerous other targets.

Pimavanserin’s lack of interaction with DA D_2_ receptors suggested that the it may be well tolerated in PDP patients receiving DA replacement therapy, A double blind, placebo controlled safety study was therefore designed in which 12 PD patients received placebo (N = 4), pimavanserin 25 mg (N = 4) or 100 mg (N = 4) once a day for 14 days [41]. The results suggested that pimavanserin was safe and well tolerated by PD patients.

We then studied the ability of pimavanserin to reduce psychosis in a Phase II study in patients with PDP. This was a double blind randomized multi-center dose-escalation study of 4 weeks duration that was designed to evaluate the safety and tolerability of pimavanserin as well as its ability to attenuate PD psychosis [42]. Patients were randomized to placebo (N = 31) or pimavanserin (N = 29). They received 20 mg of pimavanserin (or matching placebo) on day one and the daily dose could be escalated to 40 or 60 mg on days 8 and 15, respectively, based on the patients’ response to the therapy. Because of the long half-life of pimavanserin, steady state drug levels were not likely reached until day 10–14. At Day 28, the mean dose achieved for the pimavanserin arm was 44.8 mg, lower than the corresponding mean equivalent dose of 55.9 mg in the placebo arm. This was likely a result of better efficacy in the drug arm given that tolerability was similar between the arms. The primary endpoint of the study was motoric tolerability and, as expected, pimavanserin did not impair motor function compared to placebo nor did it cause sedation or hypotension. In addition, pimavanserin showed good signals of effect on the scale for assessment of positive symptoms (SAPS) hallucinations and delusions domains (SAPS H + D) and significantly attenuated the psychosis as assessed by the global item ratings of hallucinations (0.02) and delusions (0.03) and was particularly effective in reducing persecutory delusions (*p* = 0.009).

Based on these encouraging Phase II data, a large international PDP study (ACP-103-012) was initiated. The -012 study was a randomized (1:1:1), double blind, placebo controlled study in which 298 patients with PDP received placebo, 10 mg of pimavanserin or 40 mg of pimavanserin once a day for 6 weeks. The primary endpoint was attenuation of psychosis as measured by the 20 item SAPS H+D scale. Motoric tolerability compared to placebo was a key-secondary endpoint. The -012 was the largest clinical study conducted in PDP and though there were clear signals of efficacy in the 40 mg arm, an unexpectedly large placebo response (42 %) precluded statistical separation. The 10 mg pimavanserin arm showed no separation from placebo on any measure. Importantly, both drug arms confirmed the safety and motoric tolerability of pimavanserin in patients with PDP. For the 40 mg arm, additional benefits were suggested by improvements in nighttime sleep (without daytime sedation) and reduction in caregiver burden.

We had initiated a second large international PDP trial, the ACP-103-014 study, before the outcome of the -012 study was known. This study had three arms; placebo, 10 and 20 mg of pimavanserin. Just as in the -012 study, SAPS H+D was the primary endpoint and we intended to recruit 300 patients. The study was stopped early on the basis of the -012 study results and the similarities in design and lower doses used. Despite the small sample size (N = 123), the 20 mg arm showed numerical separation from the placebo arm but did not achieve statistical significance.

**Fig. 4:**
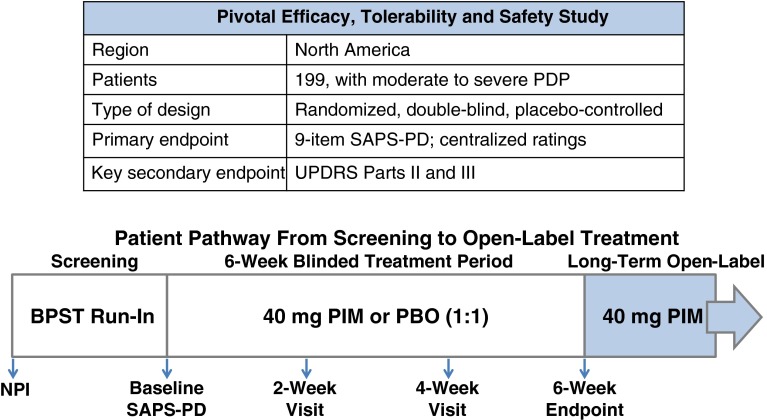
Design of the pivotal ACP-103-020 study [46]

A number of factors appeared to contribute to the high placebo response in the -012 study and were taken into account in the design of a new study, ACP-103-020 (Fig. 4) [43]. The primary endpoint, the SAPS H+D scale, had been assessed differently in the US and ex-US regions. Differences in the efficacy profile were therefore prospectively analyzed and showed a higher placebo response in the ex-US regions where site-based raters were used. In the US, a small group of well trained and independent raters interviewed patients and caregivers via a live video link. This methodology provided for lower variability and contributed to the stronger separation of the 40 mg pimavanserin arm in the US. An additional factor was that patients with milder symptoms had a larger placebo response, thus only moderately or severely psychotic PD patients were randomized to -020. Three additional design enhancements included 1:1 randomization (placebo and 40 mg of pimavanserin) and a 2-week lead-in period in which brief psychosocial therapy was offered [44]. This non-pharmacologic therapy was used prior to randomization. In addition, the nine item SAPS-PD was used for the primary endpoint, rather than the 20 item SAPS H+D. This optimized scale was developed specifically for use in PDP and eliminated items more specific to schizophrenia [45].

**Fig. 5:**
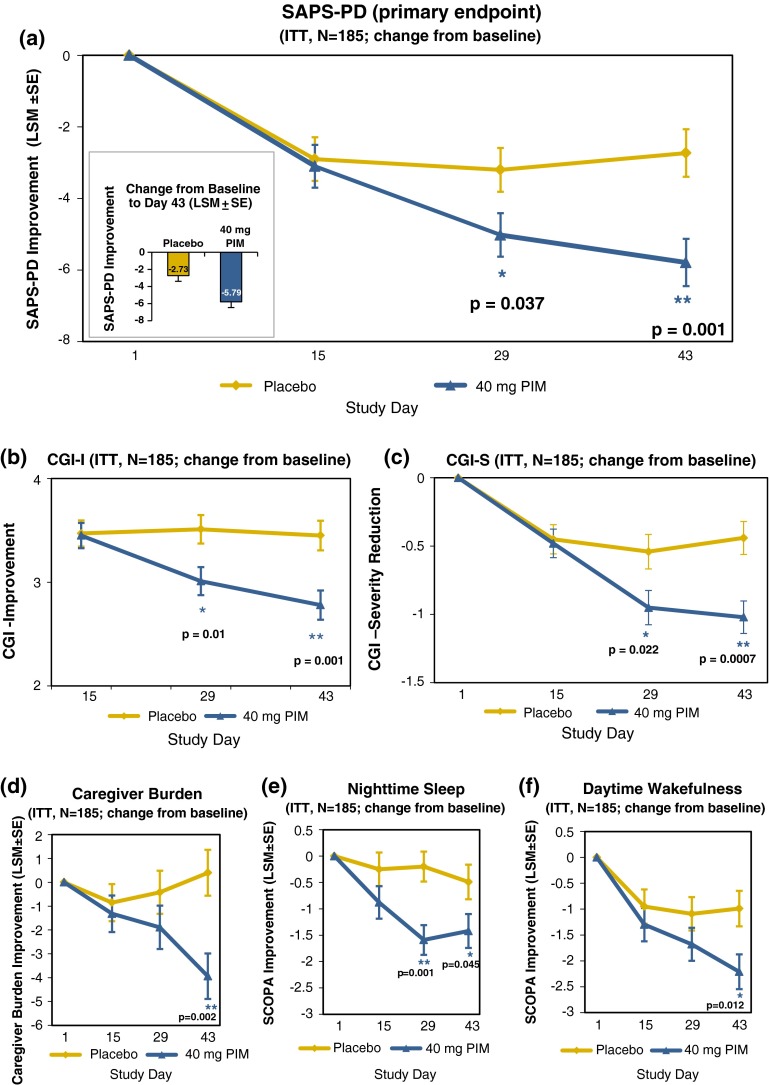
Pimavanserin 40 mg showed significant improvements over multiple endpoints measured in the ACP-103-020 study [46]. The full analysis set includes all patients who received ≥1 dose and had a SAPS assessment at baseline and at least one afterwards. Data points show least squares means (SE)

In the -020 study, pimavanserin demonstrated a highly significant and clinically meaningful improvement on the primary endpoint (Fig. 5a) [46]. As demonstrated in previous studies with pimavanserin in patients with PDP, the drug candidate was safe and did not negatively impact the motor control of the patients.

The effect on psychosis included significant improvements on both hallucinations and delusions. As this study was conducted entirely in North America, the SAPS-PD was assessed solely by blinded, independent raters. In addition, to the improvements on the centrally-rated SAPS-PD, highly significant improvements on the investigator assessed clinical global impression scale (Fig. 5b, c) and on caregiver assessed burden (Fig. 5d) scores were observed. Each of these was assessed independently such that the different raters were blind to each other’s scores. In addition, and consistent with previously observed sleep benefits in older healthy volunteers [47], patients reported highly significant improvements in nighttime sleep and daytime wakefulness using the SCOPA sleep instrument (Fig. 5e, f).

Patients who completed a blinded Phase III study had the opportunity to roll into an open label extension study. Analysis of long-term data from this study (collected up to March 2013) showed continued safety and tolerability compatible with long-term administration of the drug [48].

## Future Directions

ACADIA is now focused on finalizing the Phase III PDP program for NDA submission. Pimavanserin may also be useful for treating psychotic conditions associated with other neurodegenerative diseases and therefore a study in Alzheimer’s disease psychosis (ADP) has recently been initiated to explore this possibility. Another potential opportunity may reside in schizophrenia therapy using pimavanserin in combination with sub-therapeutic doses of atypical antipsychotic agents in order to achieve efficacy with an improved safety and tolerability profile, as suggested by the outcome of a large Phase II study in acutely psychotic schizophrenic patients [49].
